# Differing effects of attention in single-units and populations are well predicted by heterogeneous tuning and the normalization model of attention

**DOI:** 10.3389/fncom.2014.00012

**Published:** 2014-02-19

**Authors:** Yuko Hara, Franco Pestilli, Justin L. Gardner

**Affiliations:** ^1^Laboratory for Human Systems Neuroscience, RIKEN Brain Science InstituteWako, Japan; ^2^Department of Psychology, Stanford UniversityStanford, CA, USA

**Keywords:** contrast-response, spatial attention, contrast-gain, response-gain, additive-offset, efficient-selection, cueing, attention-field

## Abstract

Single-unit measurements have reported many different effects of attention on contrast-response (e.g., contrast-gain, response-gain, additive-offset dependent on visibility), while functional imaging measurements have more uniformly reported increases in response across all contrasts (additive-offset). The normalization model of attention elegantly predicts the diversity of effects of attention reported in single-units well-tuned to the stimulus, but what predictions does it make for more realistic populations of neurons with heterogeneous tuning? Are predictions in accordance with population-scale measurements? We used functional imaging data from humans to determine a realistic ratio of attention-field to stimulus-drive size (a key parameter for the model) and predicted effects of attention in a population of model neurons with heterogeneous tuning. We found that within the population, neurons well-tuned to the stimulus showed a response-gain effect, while less-well-tuned neurons showed a contrast-gain effect. Averaged across the population, these disparate effects of attention gave rise to additive-offsets in contrast-response, similar to reports in human functional imaging as well as population averages of single-units. Differences in predictions for single-units and populations were observed across a wide range of model parameters (ratios of attention-field to stimulus-drive size and the amount of baseline response modifiable by attention), offering an explanation for disparity in physiological reports. Thus, by accounting for heterogeneity in tuning of realistic neuronal populations, the normalization model of attention can not only predict responses of well-tuned neurons, but also the activity of large populations of neurons. More generally, computational models can unify physiological findings across different scales of measurement, and make links to behavior, but only if factors such as heterogeneous tuning within a population are properly accounted for.

## Introduction

Visual spatial attention is associated with a bewildering array of different effects on single neurons which appear at odds with the more uniform modulations observed from population activity such as those measured with functional imaging. Visual spatial attention, a cognitive process by which prior information about the relevance of spatial locations is used to improve perceptual performance (Noudoost et al., 2010; Carrasco, 2011), has been shown to have a variety of effects on neural responses to image contrasts (contrast-response function) in monkey visual cortex (Reynolds and Heeger, 2009). Early reports suggested that contrast-response functions shift horizontally with attention for visual area V4 (Reynolds et al., 2000; Martínez-Trujillo and Treue, 2002), termed a “contrast-gain” change. This shift of contrast-response has an appealing interpretation in that it suggests that directing attention acts much the same as if you physically increased the contrast of the stimulus at that location. Other reports, however, have favored a “response-gain” change in which there is a multiplicative change in the contrast-response function so that the largest absolute change in response occurs at the highest contrasts (Lee and Maunsell, 2010). Still others have reported additive-offsets of contrast-response dependent on stimulus visibility (Thiele et al., 2009; Pooresmaeili et al., 2010). Some experiments have even reported different effects of attention for different neurons in the same experiment (Williford and Maunsell, 2006; Reynolds and Heeger, 2009). In contrast to the diversity of effects of attention reported in single-units, multiple studies using population-scale measurements such as functional imaging of cortical activity in human visual cortex (but see Itthipuripat et al., 2014) have reported an additive-offset effect of attention in which contrast-responses increase equally with attention at all contrast levels (Buracas and Boynton, 2007; Li et al., 2008; Murray, 2008; Pestilli et al., 2011). Similar additive-offsets were also apparent in population averages of single-units (e.g., see Figure 6 in Williford and Maunsell, 2006). Computational models of attention (e.g., Itti and Koch, 2001; Boynton, 2009; Eckstein et al., 2009) offer unifying frameworks to understand various effects of attention; can the seeming difference between single-unit and population-scale response changes with attention be reconciled by theoretical predictions?

The normalization model of attention (Lee and Maunsell, 2009; Reynolds and Heeger, 2009) provides an elegant explanation for the diversity of single-unit response changes with attention. The key insight of the model is that the size of the area which a subject attends to (attention-field size) is an uncontrolled variable which can account for differences in experimental results. In particular, changes in the attention-field size can affect the divisive normalization computation which has been used to explain response-saturation (that responses do not continue to grow linearly with higher contrast: Albrecht and Hamilton, 1982) and various other non-linear properties of neurons in visual cortex (Heeger, 1992; Carandini and Heeger, 2012). When the attention-field size is small relative to the spatial extent of neural input due to the visual stimulus (stimulus-drive size), response-gain changes will dominate since the effect of attention will be strongest in the neuron well-tuned to the stimulus (E in numerator of Equation 1, Materials and Methods: Normalization model of attention) and not in the larger pool of neurons which provide the normalization signal that divisively inhibits through the denominator of the normalization equation (S in Equation 1). Conversely, for a large attention-field, the normalization signal will be large and will act to suppress responses at high contrasts, resulting in larger response-saturation and thus a contrast-gain like effect. In sum, the variety of attentional effects on the contrast-response of single-units in monkey cortex can be well explained by the model.

While the normalization model of attention provides a plausible explanation for response properties of single-units, what predictions does it make for population responses such as those measured with functional imaging? The normalization model of attention was initially developed to explain the responses of single neurons with tuning matched to the stimulus; i.e., receptive field matched both in location and feature selectivity to the stimulus (as is typically done in single-unit physiology experiments). However, neural populations have neurons with heterogeneous tuning for any given stimulus—some neurons within the population will have tuning matched to the stimulus while other neurons will, to varying degrees, be mismatched. This heterogeneity of tuning will have consequences for the predictions of the normalization model of attention since different neurons in the population will experience different balances of stimulus-drive and attention-field. For example, neurons whose receptive-field location and feature selectivity is matched to the stimulus and locus of attention will have overlapping inputs of stimulus-drive and attention-field, while neurons responding to the periphery of the stimulus may encounter larger attention-field modulations but less stimulus-drive. Likewise, the normalization pool for each of these neurons will have differing balances of stimulus-drive and attention-field. Therefore, differences in tuning properties would result in very different predictions of attentional modulation for different neurons in the neuronal population.

Here, we investigated the behavior of the normalization model of attention for neural populations with heterogeneous tuning for the stimulus. We used functional imaging to directly measure the ratio of attention-field and stimulus-drive size in human visual cortex for a contrast-discrimination task. We constrained the parameters of the normalization model based on these measurements and examined the predicted effects of attention for a population of simulated neurons as well as the summed response over the whole population. We found that under realistic parameter settings, the model could predict opposite effects of attention on the contrast-responses of different neurons in the same population; response-gain for neurons well-matched to the stimulus and contrast-gain for neurons not well-matched in either (or both) receptive field location or orientation preference. Averaging over model neurons, as is implicitly done by population-scale measurements, predominantly resulted in additive-offset of contrast-response, similar to those reported in functional imaging studies (Buracas and Boynton, 2007; Murray, 2008; Pestilli et al., 2011) and evident in averages across single-units (Williford and Maunsell, 2006). This additive-offset effect in the population average occurred across several orders of magnitude of the ratio of attention-field and stimulus-drive size. Thus, we conclude that the predictions of the normalization model for well-tuned single-units and heterogeneous neural populations can differ substantially. Furthermore, for a wide range of model parameters, the predictions of the normalization model of attention well-predict the additive effects of attention seen in measurements of human cortical population activity.

## Materials and methods

### Normalization model of attention

We used publicly available computer code from the websites of the authors of the normalization model of attention (http://www.cns.nyu.edu/heegerlab or http://www.snl-r.salk.edu/~reynolds/Normalization_Model_of_Attention). For a full description of the model and parameters, see Reynolds and Heeger (2009). The model predicts a neuron's response to visual stimuli such as oriented Gabor patches with different modulations due to spatial or feature-based (e.g., Treue and Martínez Trujillo, 1999; Liu et al., 2011) attention. In particular, we were interested in simulating model predictions of the effect of spatial attention on each neuron's response profile to different stimulus contrasts (contrast-response function). Parameters were set according to settings depicted in Figures 3D–F of Reynolds and Heeger (2009) unless otherwise noted here. In brief, each model neuron receives stimulus-drive based on a Gaussian tuning preference for both orientation (with tuning width of 60°, defined as half-width at 60.7% of maximum) and space [tuning width of 0.75° visual angle, taken from published values of population RFs measured in human V1 for the eccentricity of our stimulus (Dumoulin and Wandell, 2008)]. A small baseline activity that can be modified by attention (5e^−7^% contrast) is added to this stimulus-drive. This baseline activity increments the neuron's contrast-response to any given stimulus (regardless of contrast, and even when no stimulus is present) by adding to the actual contrast of the stimulus; thus, units are percent contrast. The response of any given model neuron is the stimulus-drive divisively normalized by a suppressive drive from similarly-tuned model neurons:

(1) $$R(x,\;\theta )=\frac{E(x,\;\theta )}{S(x,\;\theta )\;+\;\sigma }$$

where *R*(*x*, θ) is the response of a model neuron with the center of its RF located at location *x* and having preferred orientation θ. *E*(*x*, θ) is the stimulus-drive as defined above. *S*(*x*, θ) is the suppressive drive which comes from model neurons with similar tuning preference. In particular, it is the multiplication of the stimulus-drive of all model neurons with a kernel representing the spatial (Gaussian with standard deviation of 20°) and featural (all orientations) extent of the pool of model neurons contributing to the suppressive drive. σ is the contrast-gain of the model neuron and controls the left-right position of the contrast-response function. The response is thresholded to mimic the spiking threshold of neurons. In our simulation, the visual stimulus input was set to mimic the visual stimulus presented to our subjects (see below) in orientation (single orientation) and spatial extent (6° full-width). The attention-field size was set to be a Gaussian 1.4 times the size of the stimulus-drive; a factor based on our measurements of the ratio of the spatial width of the cue- and contrast-sensitivity which were taken as measures of the attention-field and stimulus-drive size, respectively (see Results: Spatial extent of stimulus-drive and attention-field in cortex). Each model neuron's stimulus-drive was multiplied by the magnitude of the attention-field, which varied according to the distance of the center of its RF relative to the center of the attention-field (located at the center of the stimulus). Population responses were obtained by averaging together all the responses of model neurons in the simulation.

To evaluate whether contrast-gain, response-gain, or additive-offsets better accounted for the predicted modulation with attention in single-unit and population responses, we asked how much of the variance in the tuning related to attention could be accounted for by these three attention effects. In particular, we fit a Naka-Rushton type equation (Naka and Rushton, 1966) of the following form to the model contrast-response functions:

(2) $$R(C)=R_{\max}\frac{C^{n}}{C^{n}+C_{50}^{n}}+R_{\text{offset}}$$

where *R* is the model response, *C* is the contrast, *R*_max_ is the maximum contrast-dependent response, *C*_50_ is the contrast at which responses reaches half maximum, n controls the steepness of the function and *R*_offset_ is the response at 0% contrast. The parameters were set to be the same for both attended and unattended model responses, except that *R*_max_ was allowed to change for the response-gain model, *C*_50_ for the contrast-gain model and *R*_offset_ for the additive-offset model. To calculate the variance accounted for by each of these three models, we computed one minus the residual variance of the model divided by the actual variance. Residual and actual variance was computed as the variance of the difference between responses in attended and unattended conditions with or without the model response subtracted, respectively.

### Human subjects

Five human subjects (ages 21–36, three male) with normal or corrected-to-normal vision participated in the study. Subjects all gave prior written consent and experimental procedures were approved in advance by the RIKEN Brain Science Institute Functional MRI Safety and Ethics Committee. Each subject participated in multiple MRI sessions including one session for acquiring a high-resolution anatomical scan, another session for retinotopic mapping and 5–7 sessions of the main experiment.

### Task

**Figure 1:**
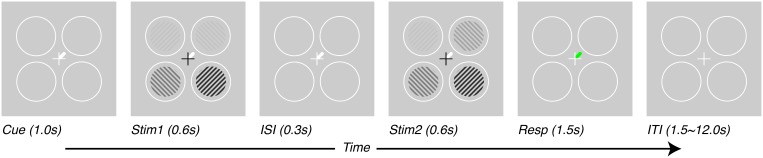
**Task design.** Subjects performed a contrast-discrimination task in one of four locations. On each trial, four contrast gratings appeared in two temporal intervals (Stim1 and Stim2) separated by an inter-stimulus interval (ISI). During one of the two intervals (Stim2 for this figure), the contrast in one location (target, upper-right for this figure) was incremented by a threshold contrast. After both stimulus presentation intervals, a green response-cue indicated the target location and subjects reported the interval during which they perceived the higher contrast with a key press (Resp). At the beginning of each trial (Cue), a white line pointed to one (or, on alternate trials, more than one) of the possible target locations, thus varying the prior information given to subjects regarding which location they would be asked to respond about. Trials were separated by an inter-trial interval (ITI) which lasted 1.5 s for tasks performed outside the scanner and 1.5–12.0 s, pseudo-randomized, inside the scanner.

Subjects performed a contrast-discrimination task as illustrated in Figure 1. Four contrast gratings (spatial frequency = 2 cycles/°, size = 3° radius, 6° eccentricity in each visual quadrant, contrasts = 12.5, 25, and 50%) were presented in two separate temporal intervals (Stim1 and Stim2, each 600 ms). All stimuli maintained the same contrast in the two intervals except one—the target stimulus, which had a slightly higher contrast in one of the two intervals. After stimulus offset, during the response interval, a green line indicated the location of the target. Subjects were asked to report the interval in which the target had the higher contrast. The difference in contrast presented between the two intervals was adjusted to a threshold level using a 1-up-2-down staircase procedure (Levitt, 1971). The target location and only the target location was shown with a contrast difference between the two temporal intervals. Cues (white line) presented at the beginning of the trial (1 s before stimulus presentation) and throughout the trial (until beginning of response interval, total duration of 2.5 s) indicated which target would need to be discriminated. Subjects were told to attend to cued locations and ignore as much as possible the other locations. In interleaved trials, the cues could point to one, two or four of the locations.

### MRI measurements and pre-processing

All MRI measurements were made with a Varian Unity Inova 4 Tesla whole-body MRI system equipped with a head gradient (now Agilent Technologies, Santa Clara, CA, USA). High-resolution 3D anatomical images were acquired with a birdcage radio frequency (RF) coil (Nova Medical, Inc., Wilmington, MA, USA) and imaging parameters were set to acquire two T1-weighted images (MPRAGE TR 13 ms, TI 500 ms, TE 7 ms, flip angle 11°, voxel size 1 × 1 × 1 mm, matrix 256 × 256 × 180) and one T2^*^-weighted image (FLASH TR 13 ms, TE 7 ms, flip angle 11°, voxel size 1 × 1 × 1 mm, matrix 256 × 256 × 180). The T1-weighted images were averaged together and divided by the T2^*^-weighted images to reduce global inhomogeneities in image contrast (Van De Moortele et al., 2009). Gray- and white-matter surfaces were generated from these images using FreeSurfer (Dale et al., 1999), from which flattened representations of the cortical surface could be constructed for visualization of data. Flattened representations were also used for defining of regions of interest which intersected with the gray-matter, such as visual areas and the regions within these areas that represented the visual stimuli in the task. Surfaces and flat maps were used only for these purposes—all data analyses and processing was conducted on the original untransformed data.

We measured blood-oxygenation-level-dependent (BOLD, Ogawa et al., 1990) signals by acquiring functional images (T2^*^-weighted) using a volume RF coil to transmit and a 4-channel receive array (Nova Medical) with an EPI imaging sequence (2-shot, SENSE acceleration factor 2, TE 25 ms, voxel size 3 × 3 × 3 mm, matrix 64 × 64). For the retinotopy experiments, 21 slices were acquired with a flip angle of 55° and volume acquisition time after SENSE acceleration of 1572 ms. For the main experiment, 16 slices were acquired with a flip angle of 51° and a volume acquisition time after SENSE acceleration of 1200 ms. Slices were oriented approximately perpendicular to the calcarine sulcus and covered early occipital visual areas. Cardiac and respiratory fluctuations measured with a pulse oximeter and a pressure sensor, respectively, were removed in post-processing (Hu et al., 1995). Motion compensation (Nestares and Heeger, 2000), linear detrending and high-pass filtering (cutoff of 0.01 Hz) were also applied. Percent signal change was computed by dividing each voxel's time-course by its mean intensity.

Retinotopic mapping (Engel et al., 1994; Wandell et al., 2007) was used in conjunction with functional localizers to separately define the locations within each visual area (V1–V3, hV4, and V3A) that responded to the stimulus (Gardner et al., 2008; for detailed procedures see: Pestilli et al., 2011). In each retinotopic scan, either a clockwise or counter-clockwise rotating wedge or expanding or contracting ring was shown for 10.5 cycles lasting 25.2 s. Typically, at least two sets of the wedge stimuli and one set of the ring stimuli were shown. After dropping the first half cycle of response, we took the Fourier transform of each response time series and determined the amplitude and phase values at the stimulus frequency (10 cycles/scan). Coherence was calculated as the amplitude at the stimulus frequency divided by the root sum of the amplitudes squared at all stimulus frequencies. Coherence and phase values were displayed on a flattened surface of the occipital cortex and visual field boundaries were drawn by hand according to published criteria (Wandell et al., 2007). In addition, we localized each stimulus within each visual field by referencing a session localizer which was run once or twice within each session. The session localizer consisted of a full contrast grating shown in sequence at each of the stimulus locations for 10.5 cycles lasting 24 s. Using coherence and phase values of the response at the stimulus frequency displayed on flattened surfaces, the location of response to each stimulus was drawn by hand. These regions were used in other analyses to report the response to each stimulus.

Event-related responses were computed for each stimulus location and each attentional condition and contrast using procedures reported in detail elsewhere (Gardner et al., 2005). Briefly, responses were computed as trial-triggered averages assuming that any response overlaps sum linearly. These event-related time series were then fit with single-gamma functions and the peak of the fit function was used as a measure of BOLD response amplitude for each trial type. The r^2^ of the model fit was used to evaluate significance of responses.

### Measuring spatial extent of stimulus-drive and attention-field

**Figure 2:**
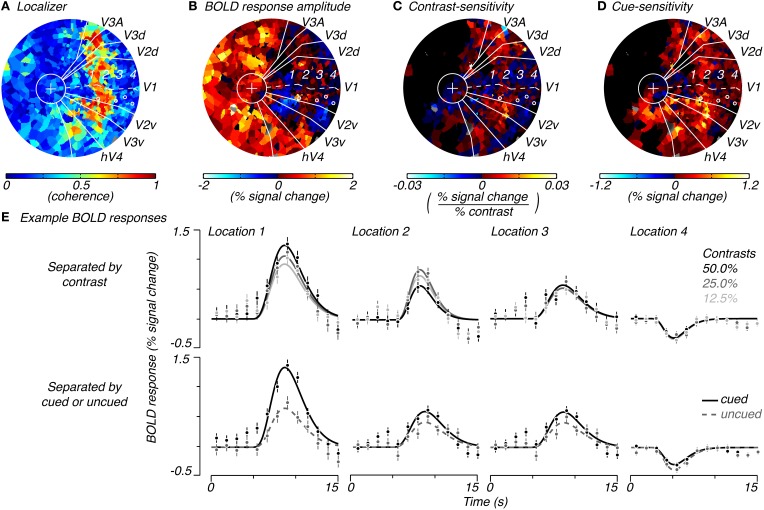
**Example of spatial characteristics of task-related signals.** Flattened representation of the occipital cortex of the right hemisphere of a single subject showing the coherence to the localizer stimulus **(A)**, average response magnitude across trials for the task **(B)**, contrast-sensitivity (**C**, slope of the contrast-response function) and cue-sensitivity (**D**, difference between BOLD amplitudes for cued and uncued locations, regardless of cue condition and pedestal contrast). Solid white lines indicate the retinotopic boundaries for V1 (a dotted line traces the calcarine sulcus), V2 (dorsal and ventral), V3 (dorsal and ventral), V3A (dorsal), and hV4 (ventral), which converge at the fovea (white circle with a “+” for fixation cross). **(E)** Shows BOLD responses for example V1 locations (white circles in **A–D**) which correspond to the 1, center, 2, edge, 3, periphery, and 4, extreme-periphery of the response to the contrast grating. BOLD responses in **(E)** are separated by pedestal contrast (top row) or by whether the response was to a cued or uncued location (bottom row). Error bars indicate standard error of mean over repeated trials.

We estimated the spatial extent of task-related attributes of measured BOLD signals—the BOLD amplitude, contrast-sensitivity, and cue-sensitivity (defined below)—by plotting these three attributes on a per-subject, per-voxel basis. These attributes were later used to estimate the spatial extent of stimulus-drive and attention-field for the normalization model. The BOLD amplitude was simply the magnitude of the average hemodynamic response elicited in that voxel averaged across pedestal contrast and cue condition. The contrast-sensitivity was the slope of the contrast-response function (in units of % signal change/% contrast) measured for each voxel (*r*^2^ > 0.7). The slope of the contrast-response function was obtained by plotting BOLD response amplitudes for each pedestal contrast, regardless of cue condition (amplitude of responses estimated from single-gamma fits to responses as shown in Figure 2E, top row), as a function of contrast (on a linear axis) and performing a linear regression. We used a linear rather than the typical sigmoid function (see Equation 2) because of the limited number of samples of contrast-response that we had acquired and because our contrast-response measurements were roughly in the range where this function appears linear. Cue-sensitivity was computed as the difference in magnitude of BOLD responses for cued and uncued conditions, regardless of the pedestal contrast or the number of cues that were used (Figure 2E, bottom row). Cue-sensitivity was positive if cueing resulted in an increase in BOLD response amplitude. The calculations for contrast- and cue-sensitivity were dependent on knowing the retinotopic location of each voxel so that the contrast and cue condition could be specified. Therefore, these attributes were calculated only for voxels in visual areas V1, V2, and V3 whose quadrant localizations could be determined.

**Figure 3:**
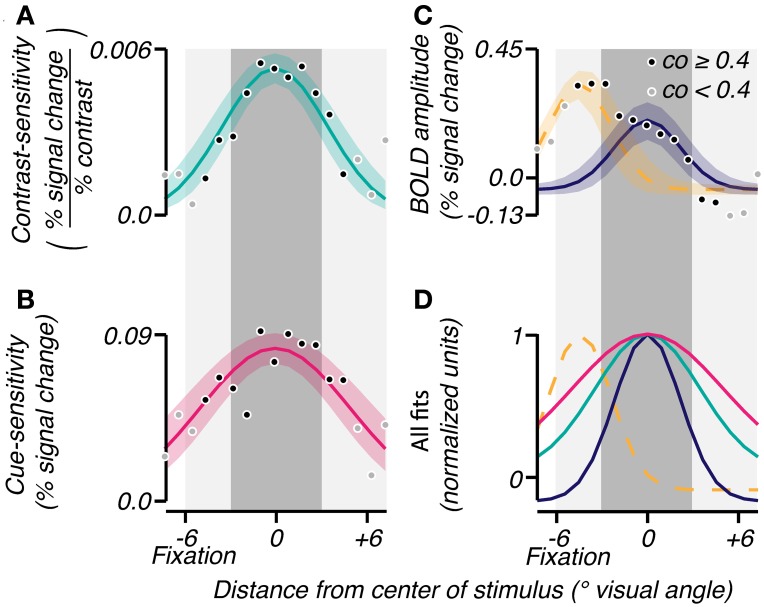
**Group analyses of spatial characteristics of task-related signals.** Gaussians were fit (bootstrap CI 95%) to the mean across subjects of contrast-sensitivity **(A)**, cue-sensitivity **(B)**, and BOLD amplitudes **(C)** binned by eccentricity. An eccentricity of 0° represents the center of the response to the stimulus as determined by the localizer. The shade of each point reflects the reliability of the eccentricity measured for that bin (coherence threshold of 0.4). In **(C)**, a sum of two Gaussians was used to account for the large activity near fixation (yellow dashed line) and the activity in response to the contrast grating (solid blue). **(D)** All fits were normalized and superimposed to visualize the range of each effect. The dark gray shaded area corresponds to the spatial extent of the contrast grating, and the light gray shaded area represents the subject's viewing range in the scanner.

The three attributes were averaged across subjects to analyze the mean extent of their effects as a function of radial eccentricity from the grating center. The grating eccentricity was estimated by averaging the eccentricities of voxels in V1, V2, and V3 whose localizer coherence exceeded 0.5. Each voxel was binned into one of seventeen eccentricity bins centered at the grating (Figure 3, 0° visual angle). The average BOLD response amplitude, contrast-sensitivity, and cue-sensitivity were calculated for each bin, resulting in sensitivity profiles as a function of radial distance from the grating center. These profiles were averaged across subjects, then fitted: the contrast- and cue-sensitivity profiles were fit with a single Gaussian (Figures 3A,B; mean fixed at 0° visual angle, offset fixed at 0, σ and amplitude were fit parameters), and the BOLD response amplitude profile was fit by a sum of two Gaussians, the first Gaussian accounting for the large response seen near the fovea and the second Gaussian accounting for the response evoked by the stimulus grating (Figure 3C; Mean of first Gaussian constrained to be < −2° visual angle, i.e., foveal to the grating location. Mean of second Gaussian fixed at 0° visual angle. Offsets, σ and amplitudes for both Gaussians were fit parameters).

We note that a similar analysis using polar angle rather than eccentricity was attempted but the nature of the topographic maps (polar angles did not offer as much cortical distance as eccentricity) and the fidelity of the topographic information (eccentricity maps were more reliable than polar angle maps) made it difficult to clearly distinguish the spatial characteristics of the various signals, especially when farther removed from the cortical locations found in the localizer.

Confidence intervals for the Gaussian fits of spatial profiles in Figures 3A–C (two-tailed, 5%) were obtained by standard nonparametric bootstrapping procedures. Responses were resampled with replacement 10,000 times, and for each iteration, the response amplitude and contrast- and cue-sensitivity profiles were recalculated and refitted with Gaussians. To compare the spatial extent of these profiles, the sigma parameter of the narrower distribution was subtracted from the sigma of the wider distribution, and a one-tailed p-value was obtained where the sorted difference changed from positive to negative (i.e., *p* < 0.0001 if the difference was positive in all 10,000 iterations).

## Results

### Measuring the spatial extent of stimulus-drive and attention-field in human cortex

We measured attention-field and stimulus-drive size in five human subjects with functional magnetic resonance imaging (fMRI) as they performed a contrast-discrimination task under different attentional cueing conditions (Pestilli et al., 2011; Hara and Gardner, 2012). Subjects were presented with four gratings of different contrasts (either 12.5, 25, or 50%) in two stimulus intervals (Stim1 and Stim2, Figure 1). After stimulus offset, a response-cue (green line, Resp interval, Figure 1) indicated a single location and subjects used a button press to report the temporal interval in which the stimulus at that location had higher contrast. To manipulate spatial attention, subjects were cued in advance (white line, Cue interval Figure 1) as to which stimuli were potentially relevant. Subjects were able to use these spatial cues to improve their behavioral performance. On average, contrast-discrimination thresholds were reduced 2.7 fold when cues pointed to a subset of locations containing the target compared to when cues pointed to all locations and provided no useful prior information (for details see Hara and Gardner, 2012).

We measured BOLD responses to these stimuli across the cortical surface and found that responses changed from positive to negative as a function of the distance from the center of stimulation. Within retinotopically-defined visual areas, we defined cortical locations that responded well to the localizer stimulus (high coherence to localizer stimulus, Figure 2A) as being within the “stimulus band.” Responses in these cortical locations showed the expected positive response (warm colors, Figure 2B and location 1, Figure 2E) with the classical hemodynamic response function shape. In cortical locations which were retinotopically more eccentric than the stimulus band, responses diminished in magnitude and, unexpectedly, turned negative (cool colors, Figure 2B and locations 2–4, Figure 2E; see Discussion: Spatial distribution of attentional signals in visual cortex for a discussion of these negative responses). We note that positive responses were also present near the fovea where the fixation cross had persisted throughout the task as well as some parts of non-retinotopically-defined cortex (Figure 2B). We interpret these to be related to fixation processes.

To measure stimulus-drive, we computed responses separately for different stimulus contrasts (Figure 2E, top row) and examined the degree to which responses were modulated by stimulus contrast. We computed the modulation to contrast as the slope of the contrast-response function and called this the voxel's *contrast-sensitivity*. Voxels with large, positive contrast-sensitivities (hot colors, Figure 2C and location 1, top row of Figure 2E) were mostly located within the stimulus band. Contrast-sensitivities decreased and approached zero outside of the stimulus band (cool colors, Figure 2C and location 3 and 4, top row of Figure 2E). Occasionally, we measured responses with a somewhat reversed relationship with contrast (smallest response with largest contrast, e.g., location 2, top row of Figure 2E) just outside the stimulus band. But these reversed responses were not consistently observed and did not come out in the group analysis (Figure 3), suggesting that they were either extremely infrequent or simply reflecting noise in the measurements.

To measure the attention-field we examined whether responses were modulated by the cue. For each voxel, we separated responses by whether the location had been cued or not cued (Figure 2E, bottom row) and took the difference in these response amplitudes and called this the voxel's *cue-sensitivity*. Cue-sensitivities were largest in voxels located inside the stimulus band (e.g., location 1, Figure 2E, bottom row) and were positive even in voxels located outside the stimulus band (Figure 2D, hot colors). Positive cue-sensitivities, therefore, were also seen in voxels whose BOLD response amplitudes were negative (e.g., location 4 in bottom row of Figure 2E). Thus, the spatial extent of positive cue-sensitivities extended well beyond areas with positive contrast-sensitivity—that is, the attention-field was larger than the stimulus-drive size.

**Figure 4:**
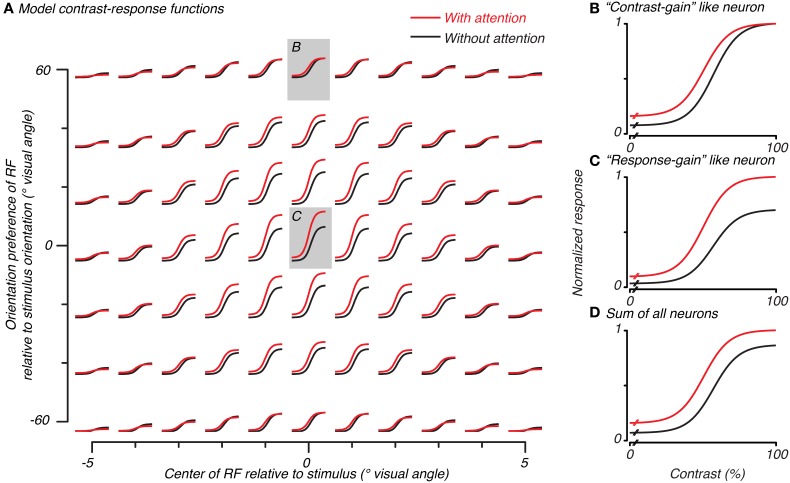
**Normalization model of attention predicts both contrast-gain and response-gain like effects, which, when summed together across space and orientation selectivity, result in an additive-offset of contrast-response with attention.** Contrast-response functions with (red) and without (black) attention for simulated neurons in the model are plotted in **(A)** as a function of the overlap of that neuron's RF (x-axis) and match of orientation preference (y-axis) with the stimulus. The central neuron's response (redrawn in **C**) had a response-gain like effect when the attention-field and stimulus-drive size ratio were set according to the measurements made to match parameters from the BOLD data. Other neurons, **(B)**, had a more contrast-gain like effect. The sum of all model neurons, **(D)**, showed a vertical shift in contrast-response with attention.

The group-averaged data confirmed the above observations, in particular, that the attention-field was larger than the stimulus-drive size. In each subject, we binned BOLD response amplitude, contrast-sensitivity and cue-sensitivity according to eccentricity. Averaging across subjects, we found that BOLD response amplitude (Figure 3C) showed peaks of activity both at the stimulus band (0° eccentricity) and near the fovea (−6° eccentricity), as described above. These were well fit by a sum of Gaussians profile (*r*^2^ = 0.93) with standard deviations of 2.17 and 2.03°, respectively. Cue-sensitivity (Figure 3B) and contrast-sensitivity (Figure 3A) were also well fit with Gaussian profiles centered at the stimulus band (*r*^2^ = 0.70 and 0.77, magenta and turquoise curves, respectively) with standard deviations of 4.92 and 3.44°, thus confirming that the attention-field was larger than the stimulus-drive size (magenta vs. turquoise line, Figure 4D; one-tailed *p* < 0.0001 on sigma values obtained on fits on 10,000 bootstrapped samples).

### The normalization model of attention predicts additive-offsets in contrast-response for population measurements

We used the cue-sensitivity and contrast-sensitivity measurements to constrain the attention-field and stimulus-drive size parameters of the normalization model of attention. In particular, we used the ratio of spatial extent of cue-sensitivity to contrast-sensitivity (4.92/3.44 ≅ 1.4, as estimated by the Gaussian fits in the previous section) as a measure of the size of the attention-field relative to the spatial extent of the feed-forward stimulus-drive in the model. We followed the approach of Reynolds and Heeger (2009), in which the model was not explicitly fit to the data, but instead, parameters were set to qualitatively reproduce experimental results in an effort to gain an intuition about its behavior. Rather than examining just the behavior of the single model neuron well-tuned to the stimulus as has been done previously (Reynolds and Heeger, 2009), we also examined neurons whose spatial (Figure 4A, abscissa) and orientation-tuning (Figure 4A, ordinate) preferences were not well-matched to the stimulus, as would actually occur in the brain, and whose response would contribute to population activity such as those measured in functional imaging.

Model neurons with receptive fields well- or poorly-tuned to the stimulus showed different types of spatial attention effects on their contrast-response functions. Well-tuned neurons showed a response-gain effect (center of Figure 4A with gray background, replotted in Figure 4C). Poorly-tuned neurons either in space or in orientation showed a more contrast-gain effect (e.g., top of Figure 4A with gray background, replotted in Figure 4B). This difference in the effect of attention can be attributed to the relative dominance of the attention-field and stimulus-drive for different model neurons. Neurons well-matched to the stimulus received a strong stimulus-drive and relatively weak normalization signal, resulting in a response-gain effect. Poorly-matched neurons received weaker stimulus-drive and larger normalization signal due to the large attention-field, resulting in a contrast-gain effect.

To determine the model prediction for BOLD measurements which explicitly average across space and implicitly over orientation preference, we examined the average response over all model neurons. This average encompassed both predictions which had response-gain effects (largest effect at the highest contrasts) and contrast-gain effects (larger effects at intermediate and lower contrasts) as well as baseline effects due to the inclusion of the modifiable baseline component which was meant to replicate baseline attention effects seen in single-unit studies (Luck et al., 1997; Reynolds et al., 2000; Williford and Maunsell, 2006). These three effects of attention summed together caused the response averaged across all model neurons to show an additive-offset effect of attention at all contrasts in the contrast-response function (Figure 4D), as has been observed in imaging experiments (Buracas and Boynton, 2007; Murray, 2008; Pestilli et al., 2011) and is evident in averages of single-unit measurements (Williford and Maunsell, 2006).

### Different predictions for well-tuned neurons and heterogeneous populations depending on model parameters

Having found that the normalization model of attention could account for additive-offsets of contrast-response across heterogeneous populations, we next examined key parameters of the model to see how robust this result was to the choice of model parameters. In particular, we examined two parameters: the ratio between the size of attention-field and stimulus-drive and the modifiable baseline response. The ratio of size of attention-field and stimulus-drive was no longer constrained to the value derived from the experimental data. Instead we tested the effect of this parameter over several orders of magnitude. Furthermore, we explored how the modifiable baseline, the amount of response added that can be modified by attention across all contrasts, affected the predicted attentional effect. The modifiable baseline allows for effects of attention when there is no stimulus, as has been seen in numerous single-unit (Luck et al., 1997; Reynolds et al., 2000; Williford and Maunsell, 2006), and functional imaging studies (Kastner et al., 1999; Buracas and Boynton, 2007; Li et al., 2008; Murray, 2008; Pestilli et al., 2011). The modifiable baseline is added to the stimulus-drive and could also be modified by attention. It is specified in units of percent contrast just like the stimulus-drive.

#### Simulating activity of well-tuned neurons

**Figure 5:**
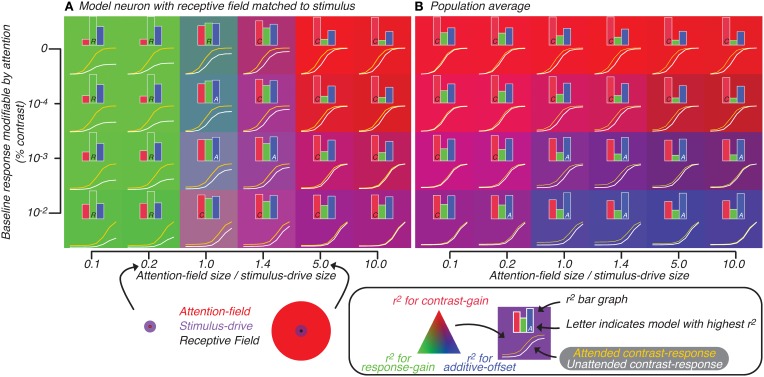
**Attention effect for best-matched neuron (A) and population average (B) predicted by model in which attention is not restricted to neurons whose tuning matches the orientation of the stimulus.** Axes represent model parameters: the amount of baseline response subject to attentional modulation (y-axis) and ratio of size of attention-field to stimulus-drive (x-axis). The ratio is visualized (bottom, along the x-axis of **A**) for cases where the size of stimulus-drive is larger (left) or smaller (right) than the attention-field size. For each set of parameters, the contrast-response function with (yellow curve) and without (white curve) attention was predicted by the model. Also for each set of parameters, a bar graph showing the degree to which each of the three attention effects models—contrast-gain (red), response-gain (green) and additive-offset (blue)—explains the variance in the tuning related to attention are shown. The model which best explains the variance is indicated by a single letter inset in the corresponding bar (C, R, and A for contrast-gain, response-gain and additive-offset, respectively). For each set of parameters, the attentional effect is also visualized by the background color (see legend below **B**).

We manipulated the above two parameters for a simulation of a model neuron with receptive field well-matched to the stimulus. Simulation results matched reports in previous work (Reynolds and Heeger, 2009) over a wide range of parameters. With no modifiable baseline (top row of Figure 5A), changing the ratio between attention-field and stimulus-drive from 0.1 to 10.0 caused the neuron with receptive field aligned to the stimulus to go from response-gain (left, compare yellow curve with attention to white curve without attention) to contrast-gain (right).

We quantified the effect of attention on the model neuron by fitting Naka-Rushton equations (Equation 2, Materials and Methods: Normalization model of attention) to the generated contrast-response functions; attention effect was modeled either as a contrast-gain change (change in *C*_50_, causes left-right shifts), a response-gain change (change in *R*_max_) or an additive-offset (change in *R*_offset_, causes up-down shifts). In Figure 5, inset histograms show for each of the three attention effects (difference between yellow and white curves) the variance that could be accounted for by each parameter alone. This analysis confirmed that as the ratio of attention-field to stimulus-drive size increased, contrast-gain rather than response-gain accounted for more of the variance. This effect was visualized (Figure 5) by setting the background color according to the *r*^2^ (see figure legend); color gradation shows clear response-gain effect (green, left side of Figure 5A) switching to contrast-gain (red, right side of Figure 5A) as the ratio of attention-field to stimulus-drive size increases.

Adding various levels of modifiable baseline (ordinate, Figure 5A), predictably increased the attention effect at low contrasts to the point that at some baseline levels, effects of attention were equally large across the whole contrast-response function, resulting in effects that appear more like additive-offsets rather than contrast-gain or response-gain like effects. In Figure 5, the inset capital letters in the bar graphs indicate which of the three attentional effects accounts for most of the variance (i.e., for conditions where the inset is a letter A, additive-offset accounted for the most amount of variance). Thus, changing the ratio of attention-field to stimulus-drive size and modifiable baseline both resulted in canonical attention effects as has been shown in the original description of the normalization model of attention (Reynolds and Heeger, 2009).

#### Simulating activity of heterogeneous neural populations

To evaluate the model predictions for population-scale measurements, we examined the average simulated activity across a large neuronal population with heterogeneous tuning. The results of this simulation on the predicted attentional effects were qualitatively very different from those for well-tuned single-units (Reynolds and Heeger, 2009). Response-gain effects were no longer found with small ratio of attention-field to stimulus-drive size (Figure 5B, left side). This was due to model neurons which were not-perfectly-tuned to the stimulus in the population whose attention effects are suppressed at high contrasts. These not well-tuned neurons receive only weak stimulus-drive regardless of the attentional condition, yet they received more divisive suppression with attention because their normalization pool included neurons whose gain had been increased by the attention-drive. Averaged across the whole population, these neurons which have reduced activity at high contrasts tend to cancel-out the response-gain of the well-tuned model neurons, resulting in a population effect that looked more like contrast-gain than response-gain.

With increased baseline effects (ordinate, Figure 5B), effects of attention are apparent across all contrast levels, resulting in effects of attention which were additive-offsets of population contrast-responses. Therefore, changes in contrast-response with attention, when averaged across heterogeneous neuronal populations, differed substantially from predictions for a single, well-tuned neuron. This can be readily appreciated by noting the overall difference in background colors in Figures 5A,B. In particular, the shift from response-gain (green) to contrast-gain (red) for the well-tuned neuron in Figure 5A is largely lost in the population averages in Figure 5B which shows predominantly contrast-gain (red) and additive-offset (blue) effects.

#### Simulating attention effects when neurons were restricted in both space and feature tuning

We further explored the prediction of the normalization model of attention by restricting attentional allocation to specific stimulus features. The population simulation described above assumes that the effect of attention is restricted in space but not to the orientation of the stimulus (i.e., the well-tuned neuron had receptive field matched to the stimulus location, not stimulus orientation). If the allocation of attention was also restricted in the feature domain, will there still be large differences in the prediction for the well-tuned single-unit and the population average?

**Figure 6:**
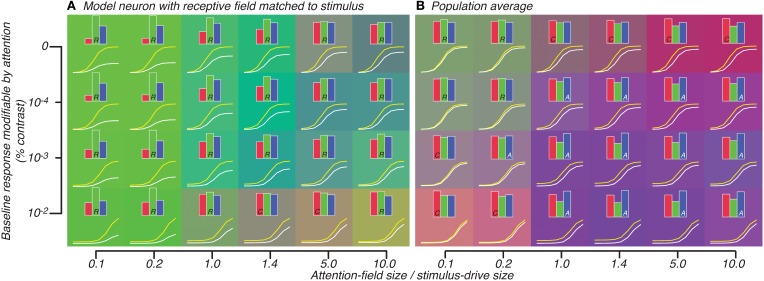
**Attention effect for best-matched neuron (A) and population average (B) predicted by model in which attention effects are restricted to neurons whose tuning matches the orientation of the stimulus.** All conventions are same as Figure 5.

We tested the effect on the well-tuned model neuron and average of the population of heterogeneously-tuned neurons when attention was also restricted along the orientation feature. In this case, the well-tuned model neuron showed response-gain effects along the whole continuum of attention-field size to stimulus-drive ratios. This is in contrast to the previous simulation in which effects ranged from contrast-gain to response-gain effects (read left-right, Figure 6A compared to Figure 5A). This was due to the suppressive-drive being effectively weaker when attention was also restricted in the orientation domain. This caused even very large ratios of attention-field to stimulus-drive size (even when ratio was 10) to still not be able to normalize responses at high contrasts. Thus, predicted attention effects across the whole set of parameters we tested were generally consistent with response-gain (note green background, Figure 6A).

Averaged across the population, however, large response-gain effects were more strongly normalized so that across a large set of parameters, the attention effect looked either like weak response-gain or additive-offset (Figure 6B). Particularly for the attention-field to stimulus-drive size ratio derived from our experimental data (1.4, 4th column in Figure 6B), attention effects were predominantly best-accounted for by additive-offsets rather than response-gain effects. This is in contrast to the response-gain effect predicted for the model single neuron which was well-tuned to the stimulus (1.4, 4th column in Figure 6A). By examining the color of the backgrounds in Figures 6A,B, one can appreciate the difference in predicted attention effects between well-tuned single-units (which largely showed response-gain effects, green, Figure 6A) and the population average (which showed mostly additive-offset effects, purple/blue, Figure 6B).

## Discussion

We have extended the normalization model of attention, which has previously been used to predict effects of attention on single neurons well-tuned for the stimulus (Reynolds and Heeger, 2009), to make predictions for populations of heterogeneously-tuned neurons. First, we used existing functional imaging data to obtain realistic estimates of the key variable of the model, the size of the attention-field relative to the stimulus-drive, in human visual cortex for a specific task and stimulus condition (Pestilli et al., 2011; Hara and Gardner, 2012). Second, we used these estimates to predict the response of populations of neurons whose tunings were not all well-matched to the stimulus spatial position and features. We found that whereas the single well-tuned neuron behaved just as described by Reynolds and Heeger (2009), other neurons in the heterogeneous population exhibited diverse attention effects. For example, while a single, well-tuned neuron showed a response-gain effect, neurons which were not well-tuned to the stimulus showed contrast-gain effects. Averaging across the entire population of neurons with heterogeneous tunings resulted in attention effects which were consistent with an additive-offset (vertical shift of contrast-response). This effect has been reported in a variety of functional imaging experiments, where measurement technique implicitly averages activity across populations of neurons (Buracas and Boynton, 2007; Li et al., 2008; Murray, 2008; Pestilli et al., 2011), and also is evident in averages of single-unit measurements (Williford and Maunsell, 2006).

We further explored the critical parameter space of the model, shifting the ratio of the attention-field to the stimulus-drive size over two orders of magnitude and adjusting the amount of baseline response subject to attentional modulation. Across these model parameter settings, we replicated model simulations showing the expected response-gain or contrast-gain effects in the single well-tuned neuron, as has been previously reported. However, we also found that when responses were averaged across the population of neurons, effects could appear significantly different from the predictions for well-tuned neurons. This was true whether we simulated attention as acting across all neurons regardless of orientation preference (Figure 5) or if we restricted attention to neurons with orientation-tuning matched to the stimulus (Figure 6). In general, these simulations show that predictions for single-units and populations can differ substantially and suggest that these potential differences must be taken into account when extrapolating model performance for single-units to population activity, particularly for models such as the normalization model of attention which were introduced to explain single-unit responses.

### Spatial distribution of attentional signals in visual cortex

In the task we studied, subjects were not explicitly instructed as to how spatial attention should be deployed. However, there were always explicit cues (circles) presented throughout the trial as to the location where targets would appear so subjects had strong prior information about how to deploy attention in a spatially specific way. Moreover, targets were always oriented gratings with the same orientation so subjects could also potentially improve performance by restricting their attention within the predictable features (orientation and spatial frequency) of the stimulus. This would be a useful strategy assuming costs to deploying attention (Attwell and Laughlin, 2001; Lennie, 2003); that is, if restricting attention modulation to the smallest possible number of neurons representing the stimulus of interest will reduce costs of attention deployment while maximizing benefits (Pestilli and Carrasco, 2005).

In previous analyses, we have found that behavioral performance in a similar attention task can be accounted for by an efficient-selection mechanism in which the primary purpose of attentional modulation is to boost signal amplitude for a pooling computation so that larger responses are weighed more heavily (Pestilli et al., 2011). In this context, a number of attentional deployment strategies might be possible. For example, subjects might try to attend to a spatial region smaller than that of the stimulus. This might have beneficial effects in that it would increase the gain of only those neurons that are tuned explicitly to the stimulus; in the efficient-selection framework, this would permit the selection of well-tuned neurons for further processing rather than neurons not-well-tuned to the stimulus. An opposite strategy would be to attend to a larger area than the stimulus. This strategy could increase the cost of attentional allocation but would benefit the subject by permitting response summation across neurons tuned to various locations and features. Our analysis of the functional imaging data provides a way to directly assess the size of attention-field relative to the stimulus-drive size and we found a ratio of approximately 1.4.

If the attention-field size is larger than the stimulus-field size, how might the brain suppress pooling of responses from the attention-field area which do not correspond to the stimulus? One characteristic of the spatial modulations across the cortex that we unexpectedly found was that responses went from positive to negative as we moved away from the center of the stimulus-response area. This mirrors so-called negative BOLD responses (Shmuel et al., 2002) in which very large stimuli result in suppression of responses outside the immediate stimulus-response area (Shmuel et al., 2006). Interpreted in the framework of our efficient-selection model, these negative responses could be another means to improve the sensory responses by suppressing pooling of responses not associated with the stimulus. The efficient-selection model weights responses according to the magnitude of response so that negative responses would not pass through the efficient-selection pooling rule. This would allow for better representation in the read-out of these visual areas, as the negative responses correspond to areas that are only weakly associated with the stimulus, yet still strongly modulated by attention.

It is possible that under different behavioral conditions, subjects may employ different attentional modulation strategies, expanding or contracting their attention-fields as needed (Eriksen and St James, 1986; Brefczynski and DeYoe, 1999; Cavanagh and Alvarez, 2005). Analyses of response modulation in functional imaging of cortical activity even suggest that humans can attend to multiple separate locations (Müller et al., 2003; McMains and Somers, 2004). With complex attentional modulation strategies, the effects predicted by the normalization model of attention could potentially be quite diverse, particularly for the effect on population-scale activity such as those measured by functional imaging. Whether response-gain, contrast-gain, or additive-offset effects dominate attentional modulations would be a complex function of the amount of attention-field and stimulus-drive that each neuron receives.

### Matching stimulus to receptive field properties

Single-unit experiments often employ an experimental strategy of matching stimuli to receptive field properties of single-units. This experimental strategy may have consequences for the interpretation as to how neural responses link to behavior as measured in such experiments. Whenever a stimulus is presented, many neurons will respond to that stimulus even if tuning properties are not particularly well-matched. Therefore, to understand computations that the brain must perform for proper perception, one needs to measure responses of neurons poorly-tuned to the stimulus as well as neurons well-tuned to the stimulus.

Our simulation of the normalization model of attention shows that under realistic ratios of attention-field to stimulus-drive size, well-tuned neurons can have response-gain effects while other neurons can show contrast-gain effects. Thus, another possible way to explain the diversity of experimental results found in the literature about single-unit changes in contrast-response with attention is that some of the variability may arise from mismatches of the stimulus properties with the tuning properties of individual neurons. Indeed, in some experiments, a continuum of changes in contrast-response were reported (Williford and Maunsell, 2006). Importantly, in this report (their Figure 6), the sum across all neurons shows effects across all contrast levels (additive-offset) much as we found for the sum across all neurons in our simulations. This is consistent with previous findings in single-unit physiology of attention which indicate that when activity is averaged across a sufficiently large and heterogeneous population of neurons, effect of attention can be seen across many contrast levels (Reynolds et al., 2000) including at baseline when no stimulus is present (Luck et al., 1997).

If attention can cause categorically different changes to response properties when receptive fields are matched or mismatched to the stimulus, how does the brain extract necessary information from a population of heterogeneously-tuned neurons? Optimal read-outs can be obtained by appropriately weighting neurons according to how much information they are expected to contain pertaining to the task at hand (Seung and Sompolinsky, 1993; Purushothaman and Bradley, 2005; Jazayeri and Movshon, 2007; Scolari and Serences, 2009, 2010; Graf et al., 2011; Scolari et al., 2012; Verghese et al., 2012). For our model, this means that predicted attention effects would dictate how neurons are weighted. For contrast discrimination, different neurons would have to be weighted differently depending on the contrast to be discriminated—for a low-contrast stimulus, a neuron with a contrast-gain effect may be more informative than a neuron with a response-gain effect. Thus, deriving optimal-weighting for neurons would require advance knowledge of the contrast of the stimulus as well as the attention effect that the neuron would undergo—each neuron can undergo either a contrast-gain or response-gain change depending on experiment conditions. According to our simulations, the type of change in contrast-response may be quite difficult to anticipate given the complex interplay between attention-field and stimulus-drive that a heterogeneous population of neurons might encounter.

## Summary

To understand how perception and behavior arise from the action of large populations of neurons, theoretical models for single-units need to be extended to encompass computational principles governing large populations. Indeed, models of population dynamics (Mante et al., 2013) or how information might be decoded from populations (Rigotti et al., 2013) offer explanation for diverse tuning properties of prefrontal neurons. In some conditions, population activity and behavioral read-outs may simply mimic the activity expected from single well-tuned neurons (Herrmann et al., 2010; Itthipuripat et al., 2014). However, our exploration of the normalization model of attention has revealed that, over a wide-range of model parameters, predictions for measures of neural activity summed across many neurons may differ substantially from those of single well-tuned neurons. Therefore, differences in predictions for population responses compared to well-tuned single-units need to be carefully accounted for when linking population activity to behavioral data through computational models.

### Conflict of interest statement

The authors declare that the research was conducted in the absence of any commercial or financial relationships that could be construed as a potential conflict of interest.
